# MicroRNA Expression Profiling of the Porcine Developing Brain

**DOI:** 10.1371/journal.pone.0014494

**Published:** 2011-01-06

**Authors:** Agnieszka Podolska, Bogumil Kaczkowski, Peter Kamp Busk, Rolf Søkilde, Thomas Litman, Merete Fredholm, Susanna Cirera

**Affiliations:** 1 Department of Basic Animal and Veterinary Sciences, Section of Genetics and Bioinformatics, Faculty of Life Sciences, University of Copenhagen, Copenhagen, Denmark; 2 Department of Biology and Biotech Research and Innovation Centre, Bioinformatics Centre, University of Copenhagen, Copenhagen, Denmark; 3 Molecular Biology, Exiqon A/S, Vedbæk, Denmark; 4 Biomarker Discovery, Exiqon A/S, Vedbæk, Denmark; Johns Hopkins University, United States of America

## Abstract

**Background:**

MicroRNAs are small, non-coding RNA molecules that regulate gene expression at the post-transcriptional level and play an important role in the control of developmental and physiological processes. In particular, the developing brain contains an impressive diversity of microRNAs. Most microRNA expression profiling studies have been performed in human or rodents and relatively limited knowledge exists in other mammalian species. The domestic pig is considered to be an excellent, alternate, large mammal model for human-related neurological studies, due to its similarity in both brain development and the growth curve when compared to humans. Considering these similarities, studies examining microRNA expression during porcine brain development could potentially be used to predict the expression profile and role of microRNAs in the human brain.

**Methodology/Principal Findings:**

MicroRNA expression profiling by use of microRNA microarrays and qPCR was performed on the porcine developing brain. Our results show that microRNA expression is regulated in a developmentally stage-specific, as well as a tissue-specific manner. Numerous developmental stage or tissue-specific microRNAs including, miR-17, miR-18a, miR-29c, miR-106a, miR-135a and b, miR-221 and miR-222 were found by microarray analysis. Expression profiles of selected candidates were confirmed by qPCR.

**Conclusions/Significance:**

The differential expression of specific microRNAs in fetal versus postnatal samples suggests that they likely play an important role in the regulation of developmental and physiological processes during brain development. The data presented here supports the notion that microRNAs act as post-transcriptional switches which may regulate gene expression when required.

## Introduction

MicroRNAs (miRNAs) are small (approximately 22 nucleotides long), highly conserved, non-coding RNA molecules that modulate gene expression at the post-transcriptional level by binding to their target mRNAs [1]. MiRNAs are believed to fine-regulate most biological processes, such as developmental timing and tissue differentiation. Moreover, miRNA deregulation is implicated in various diseases including inflammation and cancer [2]. Within the last decade, a large number of miRNAs from many different organisms has been discovered and these appear to be highly conserved across species. The most recent release of miRBase 16.0 (September 2010) [3] includes 15172 miRNA precursor entries in 142 species: 799 entries represent individual *Mus musculus* mature miRNAs and 1240 represent mature miRNAs from *Homo sapiens*. At present, only 211 mature miRNAs are annotated for *Sus scrofa* in miRBase 16.0, and more than half of these are *in silico* predictions. In the last three years, many studies on identification and characterization of porcine miRNAs have been published. A number of studies are based on cloning and sequencing [4], [5], [6], [7]. Others focus on computer predictions of miRNAs using EST or genomic sequencing data [8], [9]. Some studies use microarray profiling in order to identify miRNAs expressed in porcine tissues [10]. More recently, miRNA investigation have been performed by deep sequencing of small RNA libraries using the Solexa platform [11], [12], [13], [14]. Together, these studies have contributed substantially to increase the number of annotated porcine miRNAs within a variety of tissues. Despite these advances, this study is the first to profile miRNAs in the porcine central nervous system (CNS) by use of microarray and quantitative PCR.

The abundance and diversity of miRNAs varies in different tissues. A number of miRNAs are expressed in highly tissue- or stage-specific patterns [15], [16], while others are more broadly expressed [17], [18]. The CNS is by far the most complex organ of the mammalian body, and houses an impressive diversity of miRNAs. In fact, more than 50% of the known miRNAs have been detected in human and mouse brain [19]. Moreover, miRNAs play an important role in modulating gene expression during neuronal development, from early neurogenesis to synaptogenesis, as well as in maintenance of neuron function [20]. Dynamic changes in miRNA gene expression profiles have also been detected during brain development [21], [22]. Therefore, the CNS is a particularly interesting target for miRNA studies.

Various techniques, such as miRNA cloning, fluorescent in situ hybridization (FISH), northern blot, microarray, deep-sequencing and quantitative real-time PCR (qPCR), have been successfully used for miRNA investigation. Currently, microarrays and deep sequencing are the most commonly used techniques for high-throughput profiling of miRNA expression. Quantitative real-time PCR is an extremely sensitive technique that enables validation of selected candidate miRNAs. Both microarray and qPCR can benefit from the Locked Nucleic Acid (LNA) technology, which increases the thermal stability of the oligonucleotides [23]. These techniques have been used successfully in various studies focused on analysis of miRNA expression in the CNS particularly in model organisms, such as the mouse. In one particular study investigating miRNA expression in mouse brain, 66 miRNAs showed altered expression levels during brain development [22]. In addition, numerous miRNAs have been shown to be ubiquitously expressed in the CNS of mice [24].

The majority of the reported miRNA research has been focused on disease-related studies in either human or rodents. However, there is a need to examine miRNAs further in other mammals, such as the domestic pig. The pig brain shows more similarity to the human brain, with respect to anatomy, size, growth and development, compared to brains of other laboratory animals [25], which makes the pig an important model to be considered within biomedical sciences. Therefore, it is highly relevant to generate knowledge about miRNA gene expression in pig tissues. This would provide useful comparative information between species. One further advantage in studying the pig is that tissues from different developmental stages are easily accessible.

## Results and Discussion

In this study, expression of miRNAs in the pig developing brain was investigated by microarray analysis and subsequently validated by qPCR. Expression profiling of two distinct brain regions was performed. These regions were acquired from three different developmental stages: gestation day 50 (F50), gestation day 100 (F100) and three month-old postnatal brain (termed adult). We selected these three particular stages of development, for the following reasons: At F50, the cerebellum and cortex are in an intensive neural differentiation stage [26]; at F100, the neuronal growth spurt takes place and in a 3 month adult brain, there is active myelination and growth of glial cells [27]. Investigating different developmental time points may be useful in identifying the function of different miRNAs. Detailed studies have previously been performed in the rodent CNS [24], [28], [29], [30], although there is currently a lack of information regarding miRNA expression in the porcine CNS. The present study reveals miRNA expression patterns within the porcine developing brain and provides information on trends in the expression of particularly interesting miRNAs.

### miRCURY™ LNA microRNA Microarray

**Figure 1:**
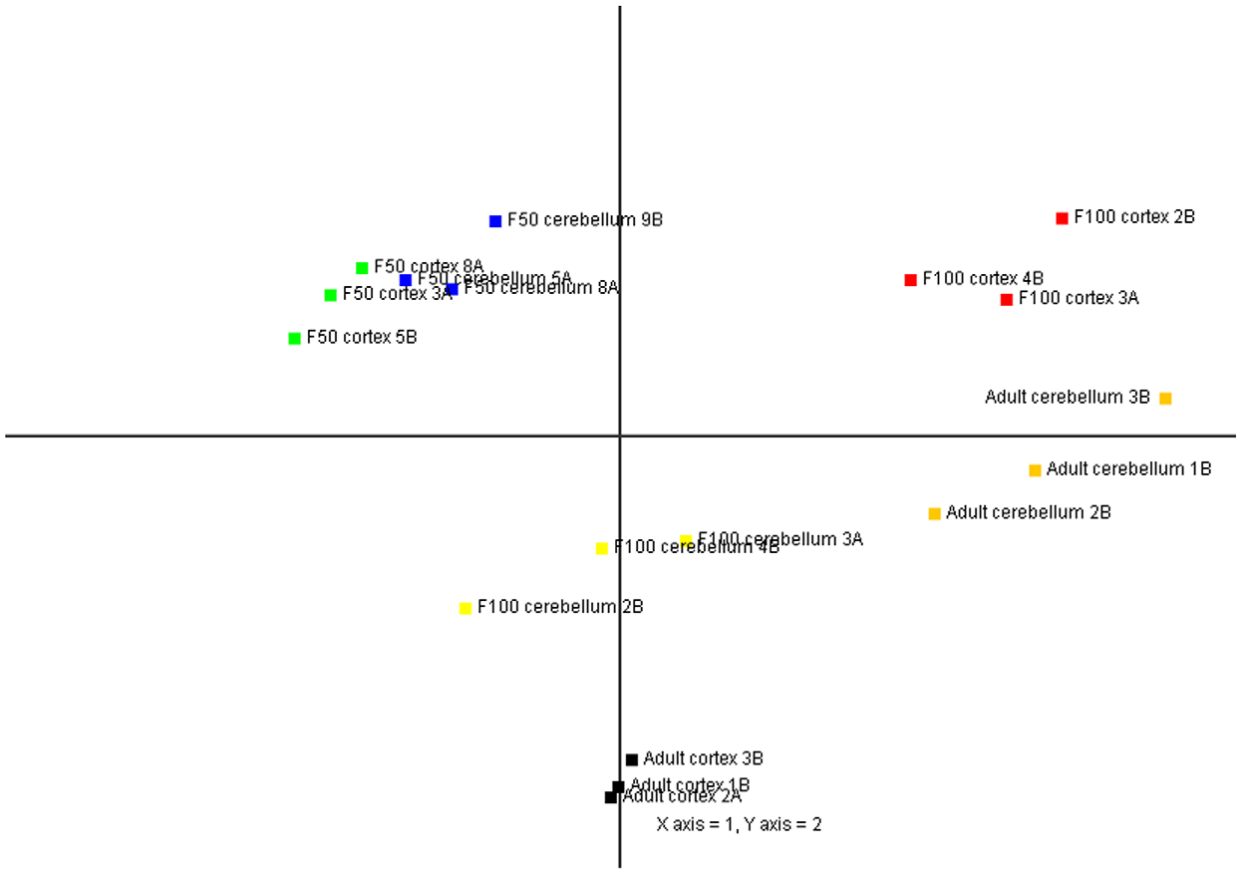
Principal Component Analysis (PCA) plot of microarray data. Clustering of the samples according to their origin is shown. In each case, three replicates from each particular developmental stage and tissue cluster together. F50 and F100 stand for fetus gestation day 50 and fetus gestation day 100, respectively.

The normalization and data filtering resulted in 1088 high quality probe signals, which represented both human and porcine miRNAs. In the initial analysis of the data, we identified general similarities and differences between the samples by means of principal component analysis (PCA). The analysis of the microarray data in Figure 1 indicated, that the miRNA expression patterns within the cortex and cerebellum from gestation day 50 (F50) were highly similar to one other. The expression patterns from the F50 samples differed considerably from the F100 and adult tissue, which suggests that changes in the global miRNA expression take place in brain between F50 and late F100 gestation. Differences between F100 cortex and adult cortex were also detected and were greater than the variation observed between the F100 cerebellum and adult cerebellum. This particular pattern may be explained by the underlying biology of brain development, with respect to different timing in the development of the cortex compared to the cerebellum during late gestation and also within the adult brain [26]. The variation in gene expression among biological replicates could originate from both technical noise and biological variation. The biological replicates of each developmental stage were highly similar in the PCA plots for both cortex and cerebellum, suggesting a low amount of technical noise and a consistent similarity in gene expression pattern among biological replicates. Additionally, the distances between developmental stages and tissue types were much larger between groups, than within groups. This illustrates that the within group variation does not pose a problem in modeling differences between the groups. Very similar microarray results were obtained from three replicates, revealing a high level of reproducibility.

**Figure 2:**
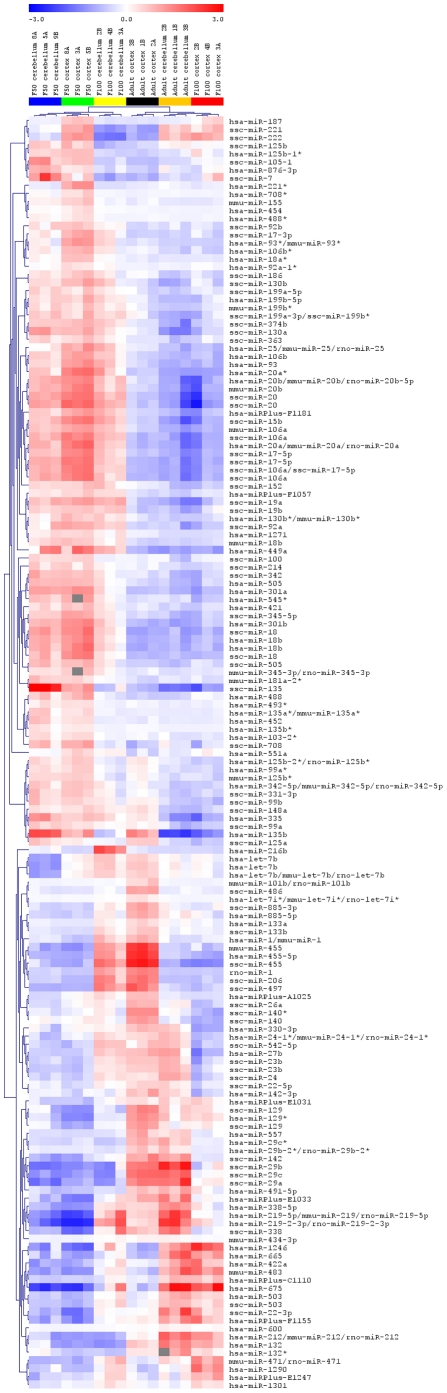
Heatmap of microRNA expression profiles in the developing porcine brain, with developmental stage being the main source of variation. Developmental stage-dependent microRNA (miRNA) expression determines the clustering. Statistically significant miRNAs (p-value <9.39E^−05^, selected by ANOVA test) are presented. Three replicates from each stage and tissue cluster together. The blue color denotes downregulated expression and the red color denotes upregulated expression level from the mean. Columns and rows represent samples and particular miRNAs, respectively. F50 and F100 stand for fetus gestation day 50 and fetus gestation day 100, respectively.

**Figure 3:**
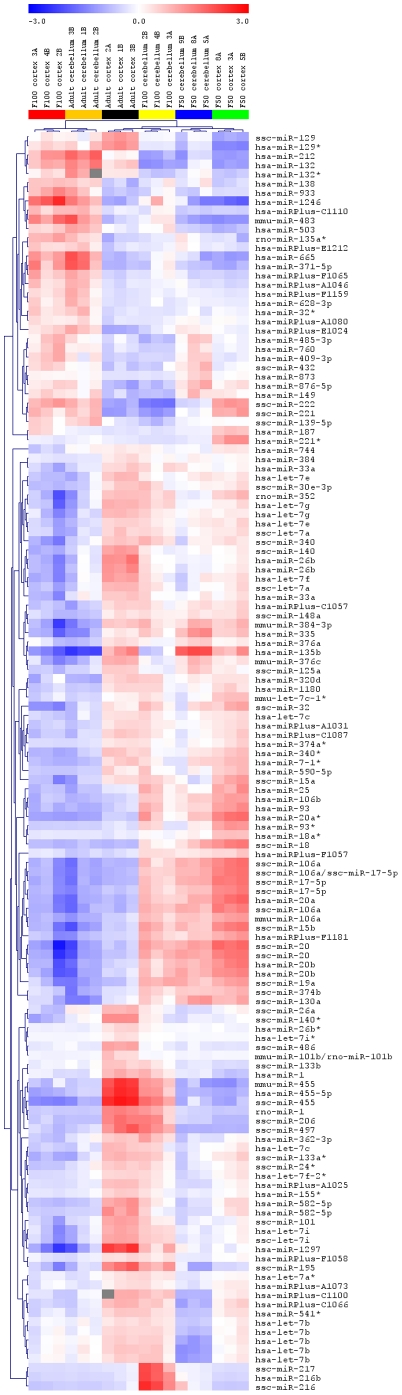
Heatmap of microRNA expression profiles in the developing porcine brain, with developmental stage as well as the tissue being the source of variation. Clustering of the samples is determined by the microRNA (miRNA) expression influenced by the developmental stage and tissue. Statistically significant miRNAs (p-value <9.95E^−06^) are presented. Three replicates from each stage and tissue cluster together. The blue color denotes downregulated expression and the red color denotes upregulated expression level from the mean. Columns and rows represent samples and particular miRNAs, respectively. F50 and F100 stand for fetus gestation day 50 and fetus gestation day 100, respectively.

Analysis of the expression of individual miRNAs revealed two different patterns of expression. The first pattern is presented in Figure 2, where developmental, stage-dependent miRNAs were used to generate the clustering. All the probes shown in Figure 2 were highly significant with p-values <9.77E^−05^. A number of miRNAs including the entire miR-17/92 cluster (with miR-17 and miR-18a being particularly significant), as well as the miR-106a/363 cluster, excluding miR-19b-2 and miR-92a-2 (with miR-106a and miR-18b having the lowest p-values), exhibited decreasing expression throughout development. The second group, e.g.; the miR-29 family (containing miR-29a –b –c) and miR-22, miR-24, miR-27b and miR-142-5p showed increasing expression with age progression, with a particularly high expression in the adult tissues. The two most interesting miRNAs within this group include the miR-221/222 cluster. This cluster had high expression within the F50 cortex and F100 cortex, compared to the adult (where high expression is detected mostly in the cerebellum). Interestingly, miR-455-3p and miR-455-5p were highly expressed in both the F100 cerebellum and the adult cortex, which suggests that the same miRNAs are expressed in both regions, but seem to reach a maximum level of expression at different time points during development. An interesting expression pattern is represented by miR-216a, miR-216b and miR-217 which were found to be exclusively expressed in the F100 cerebellum, implying that they are highly stage- and tissue-specific miRNAs. These three particular miRNAs represent very promising biomarker candidates (see Figure 3).

The expression profiles of many microRNAs were variable and distinct. This lead to the general conclusion that miRNAs are highly stage-specific regulatory elements. One striking observation was that many miRNAs exhibited significantly different expression patterns between F50 and adult brain tissue. It could be considered that miRNAs which were highly expressed in fetal tissue might be involved in the development and growth of a particular region of the brain, such as the frontal cortex or cerebellum (e.g. miR-17, miR-18 or miR-106a). Furthermore, these miRNAs might be involved in control of neurogenesis, which peaks just prior to birth. During the early postnatal period, neurons undergo terminal differentiation. Therefore, it is plausible that miRNAs prominently expressed at this time (for instance miR-24 and the miR-29 family members) might regulate this process. The cerebellum starts to develop very early in fetal life. Therefore, miRNAs up-regulated in F50 cerebellum (e.g. miR-135a, miR-135b and miR-7, see Figure 2) and F100 cerebellum (e.g. miR-216a and miR-216) make promising candidates for developmental switches in the cerebellum. The cortex is undergoing growth and intensive development at the end of gestation, as well as after birth and in youth. Therefore, miRNAs found to be over-expressed in the cortex (e.g. miR-455-3p, miR-455-5p) might be responsible for growth and neuron formation. The brain continues to grow after birth, but this primarily reflects an increase in the number of oligodendrocytes, which are responsible for brain myelination [27]. Moreover, an immense amount of new synapses are formed after birth [27]. Thus, miRNAs expressed at a high level in the adult brain (for instance let-7i, miR-22 and miR-29a-b and -c, miR-142-5p), could be involved in myelination, synapse formation and/or maintenance of synaptic plasticity.

Candidates for qPCR validation were chosen among the miRNAs that showed statistically significant differences between tissues or developmental stages. P-values for chosen candidates were as follows: miR-17(1.92E^−09^), miR-18a(2.62E^−09^), miR-29c(4.71E^−09^), miR-106a(1.84E^−08^), miR-135a(8.26E^−09^), miR-135b(1.99E^−08^), miR-221(9.19E^−05^), miR-222(2.04E^−05^) (see Supporting Table S2) Expression profiles of these candidates can be found in Figure 2. Three out of eight miRNAs exhibited decreasing expression during brain development (miR-17, miR-18a (belonging to the same cluster), miR-106a) [24]. Interestingly, miR-17, miR-18a and miR-106a belong to the same miRNA family. We also analyzed one miRNA which displayed an increase in expression during development (miR-29c). This miRNA has previously been documented to be involved in regulation of p53 expression levels [31]. The miR-135a/135b cluster was chosen because of its very interesting expression pattern in the brain and because it has not been previously described in the brain. We also analyzed the miR-221/222 cluster, due to a detectable decrease in expression within the cortex and an increase within the cerebellum during brain development.

### Validation of the miRNA candidates by qPCR

Eight selected candidate miRNAs and two miRNAs used as reference genes were assayed by qPCR in order to confirm the expression profiles found by the microarray study. Porcine miRNAs were identified based on bioinformatics and based on comparisons to corresponding human miRNA sequences. Porcine-specific LNA-spiked PCR primers were designed (Exiqon, Vedbaek, Denmark).

**Figure 4:**
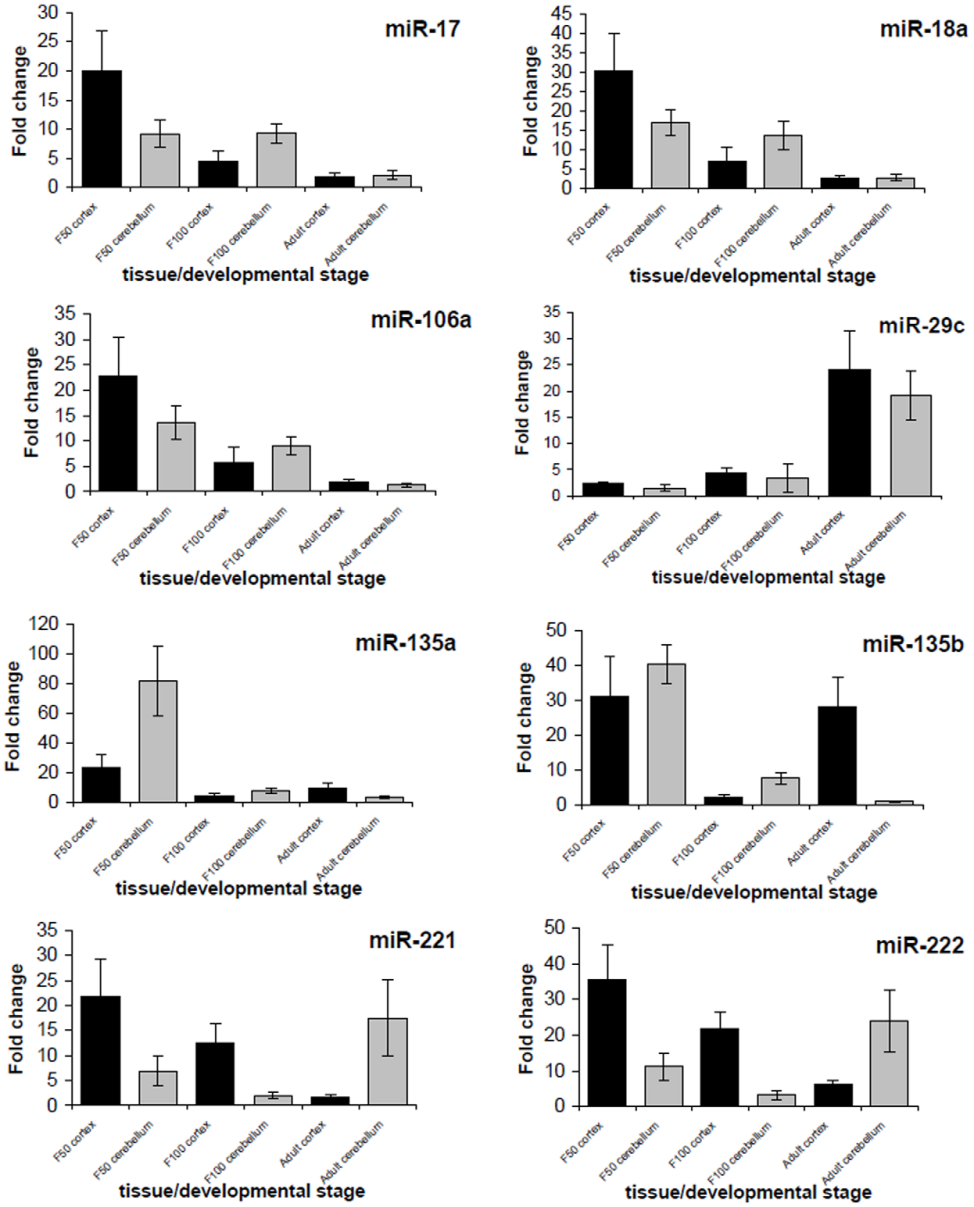
Quantitative PCR expression profiles of selected microRNAs. MicroRNA specific qPCR expression profiles. Expression rates between various samples are presented by fold changes in relation to the lowest, normalized expressed value. The error bars of qPCR represent a standard deviation of the mean of 16 replicates.

Expression profiles of each miRNA were evaluated. A one factor ANOVA test yielded P-values ranging from 9.76E^−17^ to 2.02E^−10^ which indicated that the results were statistically significant (see Supporting Table S1, and Figure 4 for specific comparisons). We could distinguish four miRNA groups which were grouped according to the temporal changes in expression.

The first group included miR-17, miR-18a and miR-106a. All three belong to the same family and miR-17 and miR-18a belong to the same cluster. These miRNAs exhibited a significant decrease in expression, which co-incided with developmental progression in the cerebellum and even more so, in the cortex. MiR-17, miR-18 and miR-106a showed up to 19, 28 and 21 fold change differences in expression in F50 versus adult tissue, with the highest level of expression observed at F50, independent of the brain tissue type (Figure 4). Co-expression of miR-17 and miR-18 has also been previously shown in rat and monkey brain, by Miska *et al*. [22]. Both miRNAs were highly expressed in the fetal tissues, followed by a decrease of the expression in the adult. MiR-17 and miR-106a play a role in the regulation of amyloid precursor protein (APP), which is known to be involved in familial Alzheimer's disease. Furthermore, a strong correlation between miR-17, miR-106a and APP has been observed in differentiating neurons and during brain development [32].

A second pattern of expression was detected for miR-29c. This miRNA increased in expression during development. Expression of miR-29c was 22- and 18-fold higher in the adult cortex and cerebellum respectively, than in the corresponding tissues at F50 (Figure 4). Higher expression in the adult brain could be associated with a potential role of miRNA in neuron maintenance or in regulation of synaptic plasticity or long-term memory. The miR-29 family (miR-29a, miR-29b and miR-29c) has previously been shown to be effective biomarkers of radiation-induced brain responses [29]. In addition, the miR-29 family is associated with the up-regulation of the tumor suppressor p53, which is central to many cellular stress responses and for inducing apoptosis [31].

A third group is represented by two miRNAs belonging to the same cluster, namely, miR-135a and miR-135b. As expected, we observed very high expression in the cerebellum at F50, followed by a dramatic decrease in expression during later brain development. A comparison between F50 cerebellum and the other tissues and developmental stages gave significant p-values (p<0.001) for both these miRNAs. In addition, miR-135b was highly expressed in the adult cerebellum. Interestingly miR-135a and miR-135b have previously been described in prostate cancer [33], breast cancer [34], as well as involved in osteoblastic differentiation (by regulating expression of bone-related genes) [35]. However, their expression profile has not been previously described in the brain.

The last two miRNAs: miR-221 and miR-222, which belong to the same cluster, constitute the fourth group of expression pattern observed. Similar to the cluster described above, these two miRNAs exhibited decreasing expression during development in the cortex and increasing expression during development in the cerebellum. It has previously been described that the miR-221/222 cluster shares a similar expression pattern in the adult hypothalamus [36]. The observed decrease of expression in the cortex of both miRNAs was found to be statistically significant with p<0.01. In the case of the cerebellum, there was no significant difference in expression in fetal tissues (F50 and F100), however, expression levels in the adult cerebellum were significantly higher (p<0.001). The miR-221/222 cluster has been found to be expressed in adult brain [30]. Moreover, Zhang *et al.*, [37] proposed that miR-221/222 may act as regulators of the tumor suppressor gene p27Kip1. When miR-221/222 expression is co-suppressed, such as in advanced human gliomas, inhibition of glioma cell proliferation by a mechanism involving the up-regulation of p27Kip is observed. In yet another study, the miR-221/222 cluster was shown to directly target the 3′ UTR regions of p27 and p57 mRNAs, which reduces reporter gene expression. Such an interaction is strongly linked to a cell cycle checkpoint that ensures cell survival, by being involved in the co-ordination of cell proliferation [38]. This might potentially explain why miR-221 and miR-222 are highly expressed in the cortex during early fetal life, when neuron terminal differentiation in the cortex and cerebellum is abundant. High expression of these two candidates in the cerebellum of the adult brain might be related to the fact that the growth spurt appears later in the cerebellum compared to the cortex, and that the cerebellum continues to develop after birth [27].

The overall expression profiling results identified a number of miRNAs that could be important for region-, or stage-specific functions within the brain. Moreover, we have revealed an interesting and novel expression pattern of the miR-135a and miR-135b cluster in the brain.

### Correlation between microarray and qPCR data

A comparison between microarray probe intensities and qPCR fold changes indicated that different miRNAs had different levels of correlation, varying from R^2^ = 0.81 to R^2^ = 0.95. The correlation coefficient values (R^2^) for particular miRNAs include: miR-17 (R^2^ = 0.84), miR-18a (R^2^ = 0.94), miR-29c (R^2^ = 0.92), miR-106a (R^2^ = 0.81) and miR-135a (R^2^ = 0.89) miR-135b (R^2^ = 0.95), miR-221 (R^2^ = 0.91) and miR-222 (R^2^ = 0.88). Thus, the overall correlation between microarray and qPCR results was extremely high. These results strengthen our reported outcomes compared to other studies, where a rather poor degree of correlation has been reported for some miRNAs [30].

### Computational prediction of miRNA targets

TargetScan was used to identify putative targets for selected miRNA candidate. In order to make the search more specific, only the most significant gene targets were taken into consideration. The same predicted targets were found for miR-17 and miR-106a. This is due to a high sequence similarity of these two miRNAs. One of the interesting targets included the myelin transcription factor 1-like (MYT1L) gene, which is considered to play a role in the development of neurons and oligodendroglia in the CNS. MiR-18a is predicted to target another CNS related gene, i.e. the neural precursor cell expressed developmentally down-regulated protein 9 (NEDD9). NEDD9 is a docking protein which plays a central co-ordinating role for tyrosine-kinase-based signalling related to cell adhesion. Interestingly, miR-135b is predicted to target a leucin zipper putative tumor suppressor 1 gene (LZTS1). This protein coding gene is ubiquitously expressed in normal tissues and is known to be involved in the cell cycle. Finally, the miR-221/222 cluster targets cyclin-dependent kinase inhibitor 1B (CDKN1B) and the tumor suppressor gene p27Kip1. The miR-221/222 cluster has previously been described to act as a regulator of p27Kip1 [37]. Computational target predictions may lead to a better understanding of the possible role of miRNAs during brain development; however, additional functional studies need to be performed, in order to confirm these predicted interactions.

### Conclusions

We have been able to identify potential developmental and stage-specific miRNA candidates, which are associated with particular functions during neuronal development and differentiation. The expression profiles established in this study provide new knowledge about gene regulation during the cerebellum and cortex development and may be valuable in comparative studies of human brain development. Additionally, we have identified the miR-135a -135b cluster in brain, which has not been previously described. These miRNAs represent potentially interesting candidates for future studies in the brain. Additional functional studies are however, required, to assess the precise role of the miRNA candidates identified.

## Materials and Methods

### Biological material

Two tissues were selected in this study, including the cortex and cerebellum. These regions were acquired from three different developmental stages: gestation day 50 (F50), gestation day 100 (F100) and three month-old postnatal brain (termed adult). Sampled tissues were immediately snap frozen in liquid nitrogen and stored at −80°C until used. For each developmental stage, four animals were used. Two replicates from each animal were submitted for RNA isolation, resulting in eight samples representing a particular tissue, at a particular developmental stage. The pigs included in this study were raised under production conditions according to Danish standards for animal husbandry. Since the animals were not subjected to experimental procedures, approval was not necessary. The pigs were euthanized by a licensed veterinarian.

### RNA extraction

The small RNA fraction was isolated from the individual tissues using the miRVana™ miRNA Isolation Kit (Applied Biosystems/Ambion, Austin, TX, USA).

100–180 mg of tissue was processed per sample, according to the manufacturer's recommendations. The resulting samples were DNAse treated with the DNA-free™ Kit (Applied Biosystems/Ambion, Austin, TX, USA). The quantity and the quality were analyzed on a NanoDrop 1000 spectrophotometer (Thermo Fisher Scientific, Waltham, MA, USA) and by visual inspection of the agarose gel electrophoresis images. Additionally, the integrities of the samples were measured by Small RNA Assay on a 2100 Bioanalyzer (Agilent Technologies, Santa Clara, CA, USA).

### miRCURY™ LNA microRNA Microarray

#### Sample preparation

250 ng of the small RNA fraction was used for each sample. Three biological replicates for each developmental stage/tissue were used for the microarray study. Samples were labeled using MiRCURY™ LNA miRNA Power labeling Kit (Exiqon, Vedbaek, Denmark), following the manufacturers' recommendations. Spike-ins (used as control probes) were added in equal amounts to each reaction and labeled.

#### Sample hybridization

Samples were hybridized to Version 11.0 arrays annotated to miRBase version 15.0, which have 1970 unique capture probes. The hybridization was performed according to the miRCURY™ LNA array manual using a Tecan HS4800 hybridization station (Tecan, Austria). After hybridization, the microarray slides were scanned and stored in an ozone- free environment (ozone level below 2.0 ppb), in order to prevent potential bleaching of the fluorescent dyes. The miRCURY™ LNA array microarray slides were scanned using the Agilent G2565BA Microarray Scanner System (Agilent Technologies, Inc., USA) and the image analysis was carried out using the ImaGene 8.0 software (BioDiscovery, Inc., USA).

### Microarray data analysis

#### Data pre-processing and normalization

The text files, generated by Imagene v.8.0, were imported into Rosetta Resolver and normalized (Rolf Søkilde - personal communication). Data were filtered by using probes showing a standard deviation (SD) above 0.1. The final, filtered data set consisted of intensity values for 1088 probes. Data are MIAME compliant and the raw data are deposited in the GEO database with accession number of: (GSE24106).

### Cluster analysis

All clustering and statistical analysis was performed in TMeV [39]. PCA plot of samples was performed using all 1088 probes, by using a median centering of the data set. For the two-way hierarchical clustering, the 181 probes on the array (which were annotated as pig miRNAs), were used. (See heatmap in Supporting Figure S1) In cases where a lack of annotation for the pig arose, then human probes were used. The 1- Pearson correlation coefficient was used as a distance metric. Each probe was mean-centered and color coded with red designating upregulation and blue designating downregulation, when compared to the mean expression.

### Data analysis

All 1088 filtered probes were used as input for a two-way ANOVA with age and brain regions used as factors. The levels for age were F50, F100 and adult, while the levels for the brain region were the cerebellum and the cortex. The full list of miRNA probes can be found in the GEO database where microarray data are submitted with an accession number of: GSE24106. The analysis was performed in TMeV and a significance cutoff p-value of less than 1.00E^−04^ was used.

There are inherently false positive discoveries in array studies. The selected cutoff of 1.00E^−04^ (false positive rate of 11%) which is a medium stringent significance filter was implemented based on our experience with miRNA array profiling, biological knowledge and the genomic context of miRNAs. For instance, the interesting candidate miR-221 which is a cluster member with miR-222 would have been discarded by using more stringent cutoff of. 1.00E^−05^ (false positive rate of 5%).

### Annotation of miRNA

Probes were first annotated to pig (ssc), and following this, other significant probes were annotated against human (hsa), mouse (mmu) and rat (rno). Some probes have multiple capture probes, as optimal probes are available for multiple species.

### qPCR

#### cDNA synthesis

Synthesis of cDNA for miRNA qPCR was performed as a one tube reaction where polyA tailing and reverse transcription was conducted simultaneously (Exiqon, Vedbaek, Denmark). Two technical replicates were used for reverse transcription, resulting in 16 cDNA samples for each particular stage and tissue. Briefly, 10 ng of the small RNA fractions was used for cDNA synthesis in a 10 µl reaction. The resulting cDNA was diluted 8 times and 1 µl was used for each qPCR reaction. Each miRNA candidate was evaluated in a total panel of 96 samples.

### Candidate genes

Eight different candidate miRNAs: hsa-miR-17, hsa-miR-18a, hsa-miR-29c, hsa-miR-106a, hsa-miR-135a hsa-miR-135b, hsa-miR-221 hsa-miR-222 and two reference miRNAs: hsa-miR-103 and hsa-miR-191 were profiled by qPCR. The choice of candidate genes was based on the statistical significance obtained following an ANOVA test of the microarray results (see results section). The choice of reference genes was based on recommendation from the literature [40].

### qPCR reaction

One qPCR reaction per sample for each miRNA candidate was performed, using 5 µl of QuantiFast SYBR Green PCR master mix (Qiagen, Germany), 0.25 mM of each primer, 1 µl of 8x diluted cDNA, milliQ water up to 10 µl total reaction volume. PCR amplification was performed in white PCR plates (ABgene, Epsom, UK); on an MX3000P machine (Stratagene, Le Jolla, CA, USA). The cycling conditions were: 1 cycle at 95°C/10 min, followed by 40 two-segment cycles of amplification (95°C/30 sec, 60°C/60 sec. The fluorescence was automatically measured during the PCR, and one three-segment cycle of the product melting curve was performed (95°C/1 min, 55°C/30 sec, 95°C/30 sec). Mx3000/Mx Pro software was used to construct a melting curve. Standard curves with 5 fold dilutions (made from the pool consisting of equal amounts of the 96 cDNA samples), were performed for each assay and the PCR efficiency calculations were based on the slopes of the standard curves. Quantification cycle (Cq) and initial template quantities for each reaction were determined with use of the baseline adjustment method from the Mx3000/MxPro software (Stratagene, Le Jolla, CA, USA).

### qPCR data analysis

Quantities of each sample were used for the analysis. GeNorm software was used to analyze the stability of the reference genes: miR-103 and miR-191. An M value below 1.5, which represents gene stability, was considered as acceptable. The two reference genes, namely miR-191 and miR-103 (M value for both was 0.787), were used for calculation of normalization factors (NF). Samples were normalized by the NF and fold changes were calculated in relation to the lowest, normalized expressed value, and then tested for normal distribution by GraphPad InStat 3 software. The one factor Analysis of Variance (ANOVA) statistical test was performed to test for significant differences between the means of the analyzed groups (for detailed comparisons see Supporting Table S1).

## Supporting Information

Figure S1 Heatmap of microRNA expression profiles for all high quality probes. Heat maps showing relative expression values of all high quality probes. F100 refers to fetus gestation day 100. F50 refers to fetus gestation day 50. The blue color denotes down regulation expression and alternately, the red color denotes up regulation expression levels above the mean. Columns and rows represent samples and particular microRNAs, respectively.
(9.10 MB TIF)

Table S1 The results of Tukey-Kramer multiple comparisons test between each sample group representing particular developmental stage and tissue. One-way ANOVA was applied to determine the probability values presented.
(0.02 MB XLS)

Table S2 The list of probability values following ANOVA analysis of the microarray data, resulted in a number of microRNA (miRNA) candidates chosen for qPCR validation. P-values for miR-221/222 were obtained when both stage and tissue was considered statistically variable. Values for other miRNAs were obtained when the developmental stage was the considered variable. Ssc and hsa stand for Sus scrofa and Homo sapiens probe, respectively.
(0.02 MB XLS)
