# Supplementary figures and images for: MicroRNA Expression Profiling of the Porcine Developing Brain
Source: PLoS One. 2011 Jan 6;6(1):e14494. doi: 10.1371/journal.pone.0014494 (PMC3017054; doi:10.1371/journal.pone.0014494)

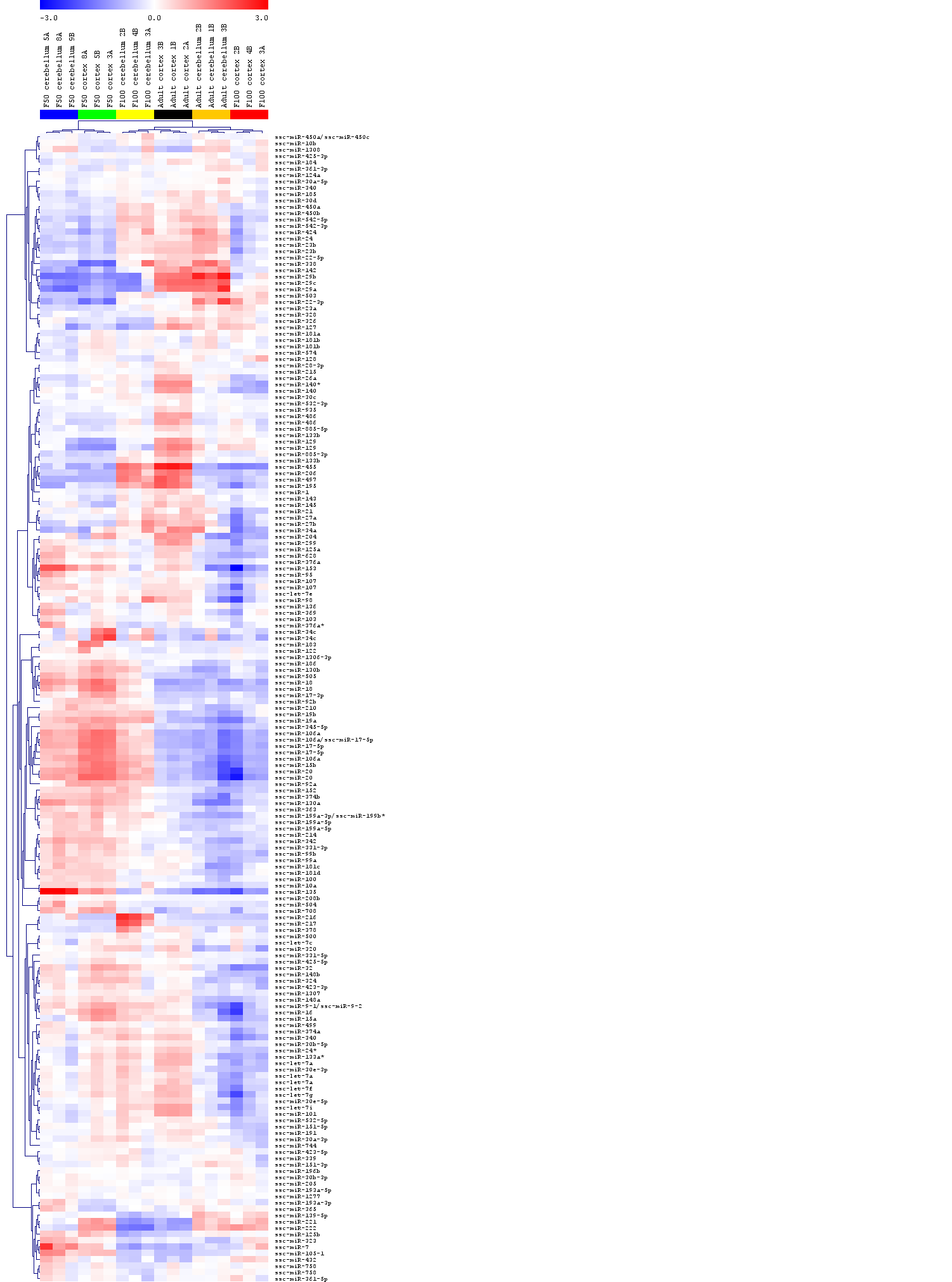

Supplement: Figure S1 — Heatmap of microRNA expression profiles for all high quality probes. Heat maps showing relative expression values of all high quality probes. F100 refers to fetus gestation day 100. F50 refers to fetus gestation day 50. The blue color denotes down regulation expression and alternately, the red color denotes up regulation expression levels above the mean. Columns and rows represent samples and particular microRNAs, respectively. (9.10 MB TIF) [file pone.0014494.s001.tif]
